# How much is sexual minority stress confounded with familial causes? A systematic multilevel meta-analysis

**DOI:** 10.1017/S0033291726105546

**Published:** 2026-08-10

**Authors:** Péter Przemyslaw Ujma, John D. Haltigan, J. Michael Bailey

**Affiliations:** 1 Institute of Behavioural Sciences, Semmelweis University: Semmelweis Egyetem, Hungary; 2 Center for Heterodox Social Science, The University of Buckingham, UK; 3 Department of Psychology, Northwestern University, USA

**Keywords:** confounding, genetics, minority stress, mental health disparities, meta-analysis, sexual minority stress, sexual orientation

## Abstract

**Background:**

Compared with heterosexual persons, non-heterosexual persons have worse mental health. Sexual Minority Stress Theory (SMST) explains the disparity as resulting from stigma and discrimination. To the extent that heterosexual genetic relatives reared with non-heterosexual persons also have worse mental health, SMST is falsified. We conducted a systematic meta-analysis of studies containing family-genetic comparison data to interrogate the empirical support for SMST.

**Method:**

We systematically identified 17 empirical studies in which twins or non-twin siblings reported on both their sexual orientation and their mental health. Subsequently, we conducted a multilevel meta-analysis, focusing on the degree to which any sexual orientation disparity in mental health diminished with genetic relatedness.

**Results:**

Most of the mental health disparities between non-heterosexual and heterosexual persons were eliminated after controlling for family-genetic factors.

**Conclusions:**

The mental health disparity between non-heterosexual and heterosexual persons is reduced by up to two-thirds once familial background factors are accounted for by comparing monozygotic twins discordant for sexual orientation. This suggests that shared familial causes, and not minority stress, are the most important causes of mental health disparities in non-heterosexual persons.

## Introduction

Homosexual and bisexual persons report worse mental health compared with heterosexual persons, on average (Bailey, 1999; Cochran et al., 2003; Lucassen et al., 2017; Meyer, Dietrich & Schwartz, 2008; Plöderl & Tremblay, 2015; Semlyen, King, Varney & Hagger-Johnson, 2016; Williams et al., 2022). Well-replicated differences include depression, anxiety, and suicidality. They have been demonstrated both for studies using formal diagnostic criteria as well as those using continuous, dimensional global mental health scales. Disparities have been regularly found for both males and females across a wide age range and in different nations.

### Sexual minority stress theory

The most influential explanation of the mental health disparities between heterosexual and non-heterosexual persons has emphasized minority stress (Brooks, 1981; Frost & Meyer, 2023; Meyer, 2003). Sexual Minority Stress Theory (SMST) proposes that “[mental health] disparities are produced by excess exposures to social stress faced by sexual minority populations due to their stigmatized social status (relative to heterosexual populations)” (Frost & Meyer, 2023). This social stress includes both structural aspects (e.g. hiring discrimination) and interpersonal aspects (e.g. rejection and bullying).

SMST’s emphasis on the importance of social stress seems plausible. For well over one thousand years, homosexuality has been stigmatized in Western civilization, sometimes severely (Crompton, 2003). However, this stigma has markedly diminished during the past few decades (Herek, 2010). These attitudes have been systematically assessed in the World Values Survey since 1984, when 75% of Americans opined that “homosexuality can never or rarely be justified.” Even in the most tolerant country surveyed that year, the Netherlands, 34% of respondents agreed with that statement. In the most recent available data, from 2022, these figures declined substantially to 28% and 6%, respectively. The Pew Research Center has found similar attitudinal changes. The percentage of U.S. respondents agreeing that homosexuality should be accepted by society increased from 51% in 2002 to 72% in 2019. The Netherlands was only surveyed in 2019, with 92% agreement. (Although survey results outside of the Industrialized West often reveal more negative current attitudes, empirical research on the SMST has been conducted almost entirely in the West.)

Surveys of non-heterosexual persons are consistent with these changes but also suggest there is considerable room for improvement. In a 2025 survey of 3,959 U.S. adults recruited for having “LGBTQ” identification (87% of whom identified as “gay,” “lesbian,” or “bisexual”), 61% agreed that there is “a fair amount of acceptance for people who are gay or lesbian” (Pew Research Center, 2025a). However, 73% of gay or lesbian adults reported having been subject to slurs or jokes because of their sexual orientation (22% in the year prior to the survey). About half said they had feared for their personal safety at some point.

#### Are sexual orientation disparities in mental health caused by minority stress?

Non-heterosexual people’s experiences of prejudice and worse mental health are empirical, factual observations that motivate SMST, not evidence for it. What is the evidence for SMST’s essential hypothesis: that differences in sexual minority stress cause the mental health disparities? Like all research on the origin of mental health problems, establishing causation is complicated by the fact that randomized controlled experimental studies (RCTs) cannot be conducted. Instead, observational studies evaluate theories with respect to their compatibility with data. Across a number of studies whose limitations vary, theories become more or less plausible. Importantly, advancing one causal hypothesis benefits from showing its empirical superiority to another.

The SMST literature is daunting in quantity. An early paper by its most prominent proponent (Meyer, 2003) has been cited more than 20,000 times as of January 17, 2026, according to Google Scholar. Despite this output, we have found few studies relevant to fundamental causal issues. Instead, one common goal of SMST research has been to show that non-heterosexual persons’ experiences related to minority stress partly statistically mediate the association between sexual orientation and mental health (Argyriou, Goldsmith & Rimes, 2021). However, statistical mediation studies cannot establish actual causation (Fiedler et al., 2011). Rather, they provide evidence regarding the degree of influence that a hypothesized mediator might have, if we assume that it is in fact a cause and one knows the correct causal model. This is true even for experimental studies (Green, Ha & Bullock, 2010) and longitudinal observational studies (Rohrer & Murayama, 2023).

To be sure, some quasi-experimental observational studies have capitalized on geographical variation in attitudes toward sexual minorities, laws protecting their rights, and relevant policy changes (Hatzenbuehler, Keyes & Hasin, 2009; Hatzenbuehler, McLaughlin, Keyes & Hasin, 2010; Pachankis et al., 2021; Perales & Todd, 2018; Raifman, Moscoe, Austin & McConnell, 2017; Raifman et al., 2020). Results from these studies have sometimes indicated that sexual minorities in more tolerant locations report better mental health compared with those in less tolerant places. These quasi-experimental studies are recent and rare, and thus have not yet been systematically replicated in a rigorous manner (e.g. preregistration). Even assuming the associations are robust, plausible alternative explanations must be empirically tested and ruled out. These include selective migration (e.g. mentally healthier homosexual persons are more likely to move to tolerant locations) and demand characteristics (e.g. homosexual respondents’ resentment of less tolerant local laws affecting their survey responses).

#### Challenges to SMST

Recent critiques have illuminated evidence that challenges SMST (Bailey, 2020, 2021; Ujma, 2022a, 2022b). This evidence includes two general categories of findings: violations of minority stress predictions and confounds that might account for associations between sexual orientation and mental health.

*Violations of minority stress predictions.* SMST is a special application of Minority Stress Theory, which applies to other groups who have experienced discrimination and intolerance. A recent survey of Black Americans reported experiencing high levels of discrimination, with 90% reporting at least “some” and 70% “a lot” of discrimination (Pew Research Center, 2025b). Yet this group does not have higher rates of depression and anxiety compared with White Americans (Ettman et al., 2022). Black Americans have lower rates of the most common anxiety disorders compared with White Americans, with the exception of PTSD (Asnaani et al., 2010; Himle et al., 2009). Furthermore, Black Americans report lower rates of suicidal ideation but higher rates of suicide attempts (Bommersbach, Rosenheck & Rhee, 2023). These patterns differ from those for sexual orientation, in which non-heterosexual persons tend to show higher rates of all these problems compared with heterosexual persons. Thus, they are difficult to reconcile with Minority Stress Theory.

Two obvious predictions exploit the considerable geographic and cohort variation in social acceptance of sexual minorities: Non-heterosexual persons in locations (e.g. nations) with higher acceptance should have lower rates of mental illness symptomatology, and there should be decreasing rates in recent Western cohorts, as acceptance has grown. Both predictions have been clearly violated in some empirical studies.

A recent review of international studies, which found very high rates of mental disorders among sexual minorities, failed to find the expected negative association between acceptance and rates of mental disorders among non-heterosexual persons (Johnson, Bogdanova, Alexander & Stokes, 2025). Indeed, the only relevant statistically significant finding in the study (based on an analysis restricted to North America) was unexpectedly positive. Regarding trends over time, not only has research failed to demonstrate robust improvement in sexual minority mental health, but several studies have also shown a worsening of mental health outcomes (Bai et al., 2025; Liu et al., 2021; Lynch et al., 2020; Meyer et al., 2021; Raifman et al., 2020).

*Confounds.* In the general population, factors other than stigma and discrimination are important causes of mental health problems. For example, temperament (i.e. early emerging behavioral and psychological traits) predicts, and may cause, later psychopathology (Tackett, 2006). Foremost among these traits is neuroticism (Lahey, 2009). Signs of a neurotic temperament often emerge quite early, well before puberty when sexual orientation becomes apparent for most persons. Genetic factors, which are important causes of both mental health problems and temperament (Flint & Kendler, 2014; Polderman et al., 2015), preexist life experiences.

SMST research has ignored a possible role of genetically influenced temperament. There is evidence, however, for early emerging temperamental differences between heterosexual and homosexual persons (Bailey, 2020, 2021). Furthermore, genes for non-heterosexuality are positively correlated with those for major depressive disorder and neuroticism, for both males and females (Ganna et al., 2019).

#### Current status of SMST

Despite considerable research on SMST, evidence for its importance remains correlational. Nor has this research addressed major threats to the theory, especially the possibility that preexisting temperamental factors correlated with sexual orientation cause (or predict) mental health disparities between individuals discordant for heterosexuality.

The challenges to SMST that we have reviewed do not falsify the theory. For example, findings that violate SMST might be explicable if the effects of minority stress varied between cohorts and geographic locations, but not within them. Genetic correlations between distress and sexual orientation could arise through many different pathways, including minority stress.

SMST research to date suffers from a lack of *strong inference* (Platt, 1964). Strong inference characterizes rapidly advancing research and includes the following practices: seriously considering alternative hypotheses, devising one or more crucial experiments (not necessarily RCTs) to falsify or confirm these alternatives, and rigorously performing the crucial experiments. All three practices have been missing from SMST research.

#### A way forward with strong inference: analysis of genetically informed samples

Consider monozygotic (MZ) twin pairs discordant for sexual orientation; that is, genetically identical twin pairs with one non-heterosexual twin and one heterosexual twin. By SMST, the non-heterosexual twins should have elevated rates of mental illness symptomatology than their heterosexual twins, and the difference should be equal to the difference seen in the general population (i.e. without comparing family members). Conversely, if the association between non-heterosexuality and mental illness symptomatology and/or disorders reflects shared familial causes – most likely, a shared genetic background – then we would expect the heterosexual cotwins of non-heterosexual twins also to have elevated mental illness symptomatology. Heterosexual twins’ mental illness symptomatology can range from similar to genetically unrelated controls (consistent with SMST) to the same level as their non-heterosexual twins (falsifying SMST). The intermediate case, in which heterosexual twins have elevated mental illness symptomatology but less than their non-heterosexual twins, would show that sexual minority stress cannot account for the entire association between mental illness symptomatology and sexual orientation, but might account for some of it.

In the current study, we systematically identified 17 empirical studies in which twins or non-twin siblings reported on both their sexual orientation and their mental health. Subsequently, we conducted a multilevel meta-analysis, focusing on the degree to which any sexual orientation disparity in mental health diminished with genetic relatedness. SMST predicts no reduction. Any reduction falsifies SMST, at least with respect to the reduction.

## Method

### Search strategy

Inclusion criteria were studies published in English using genetically informed designs (e.g. co-twin or sibling control) to report full-population (non-family clustered) and within-family mental health differences as a function of sexual orientation. PubMed, Scopus, and PsycNET were searched with the following search terms (limited to the Title and Abstract of studies) to find studies for inclusion in the analyses:

(“sexual minority” OR LGBT OR gay OR lesbian OR bisexual OR “sexual orientation”) AND (twin OR sibling OR genetic) AND (“mental health” OR psychiatric OR depression OR anxiety OR distress OR stress OR suicide)

**Figure 1.**
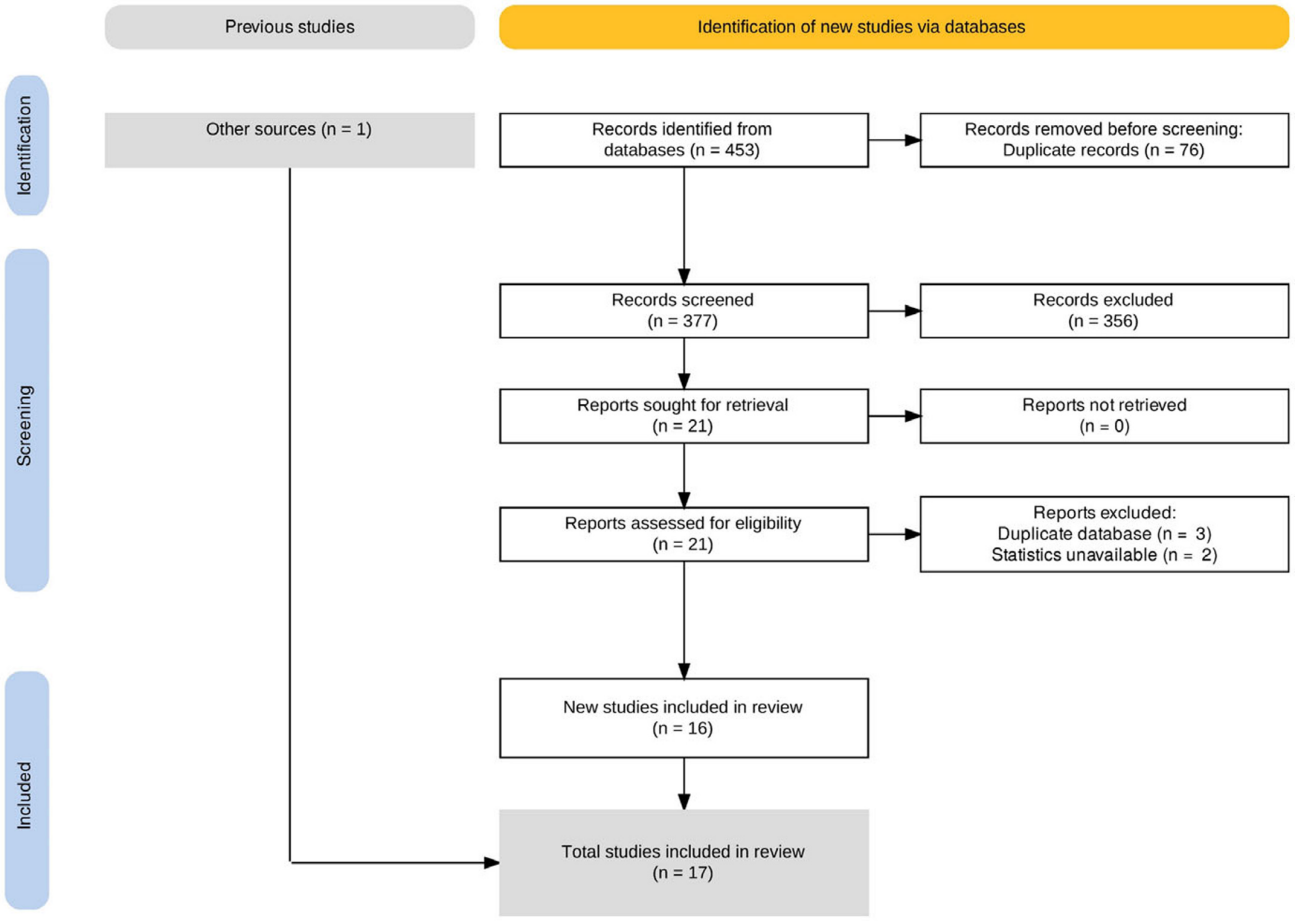
PRISMA workflow chart of the literature search. Figure 1. long description.

The literature search identified 453 records, of which 16 were included after screening (Bränström, Hatzenbuehler, Tinghög & Pachankis, 2018; Bränström, Narusyte & Svedberg, 2023; Donahue et al., 2017; Frisell, Lichtenstein, Rahman & Långström, 2010; Herrell et al., 1999; O’Reilly et al., 2021; Oginni et al., 2023a, 2023b; Post et al., 2021; Rosario, Reisner, Corliss, Wypij, Calzo, et al., 2014a; Rosario, Reisner, Corliss, Wypij, Frazier, et al., 2014b; Sánchez, Bocklandt & Vilain, 2013; Timmins, Rimes, & Rahman, 2018; Xu, Rahman, Hiyoshi, & Montgomery, 2023a, Zietsch et al., 2011, 2012). One additional study (Marzecki et al., 2024) was added from a personal collection. Three studies (Oginni, Jern & Rijsdijk, 2020; Oginni et al., 2022a, 2022b were screened as full texts but excluded because they analyzed datasets also available in other included studies. Two studies (Balsam, Beauchaine, Mickey & Rothblum, 2005; Rothblum & Factor, 2001) were screened as full texts but excluded because statistics were reported in insufficient detail. Figure 1 presents the literature search workflow. Study screening was completed in Rayyan.

The co-sibling analysis, one of two analyses presented in one paper (Xu, Rahman, Hiyoshi, & Montgomery, 2023a), was considered separately, for several reasons. Most importantly, a lack of comparable reported statistics (hazard ratios instead of odds ratios reported) rendered it challenging to integrate it in a meta-analytic synthesis of the literature, while its operationalization of sexual orientation (being in a same-sex versus opposite-sex marriage) was unique (see Supplementary material and commentaries [Sanders, 2023; Xu, Rahman, Hiyoshi, & Montgomery, 2023b; Zietsch, 2023] concerning the strengths and limitations of this study). However, because this analysis is larger than all the others combined and reports a smaller than usual reduction in effect sizes in within-family analyses, we report exploratory analyses with the inclusion of this study.

### Data analysis

For each study, the following were extracted: effect sizes (in Cohen’s *d* units) representing a difference in mental health, for up to three kinds of comparisons. First, all heterosexual and non-heterosexual persons in the sample were compared, without considering family clustering. Second, across all pairs of family members discordant for sexual orientation, heterosexual and non-heterosexual persons were compared. Third, if sufficient information was available, heterosexual and non-heterosexual members of MZ twin pairs discordant for sexual orientation were compared. Although the comparison of MZ twin pairs is the most powerful way to control familial confounding, those estimates were reported in only a minority of studies. Depending on the extent of information relevant to mental health, multiple effect sizes were extracted for each comparison.

Because the “any family” specification includes both DZ/sibling and MZ comparisons, it is expected to account for between 50% and 100% of familial effects, while the “MZ twin” specification accounts for 100%, albeit with less precision. A random-effects meta-analysis was performed using the metafor package in R (Viechtbauer, 2010), with random intercepts for studies. Comparison type (familial effects uncontrolled, any family control, MZ twin control) was used as a moderator. Detailed information about calculation methods is available in the Supplementary material. Raw data, literature search details, a documentation of our Rayyan workflow, and code are available at https://osf.io/jwhdm/.

## Results

The proportion of within-study variance was relatively low at 3.3%, with substantial residual heterogeneity (*Q* = 832.6, *p* < 0.001). The estimated mental health disparity in uncontrolled population estimates (i.e. the comparison) was *d* = 0.408 (*SE* = 0.045, *p* < 0.001). Publication bias was checked via Egger’s test after averaging effect sizes within the same study. There was borderline significant publication bias in population estimates (*z* = 2.08, *p* = 0.04) but not in the within-family estimates.

**Figure 2.**
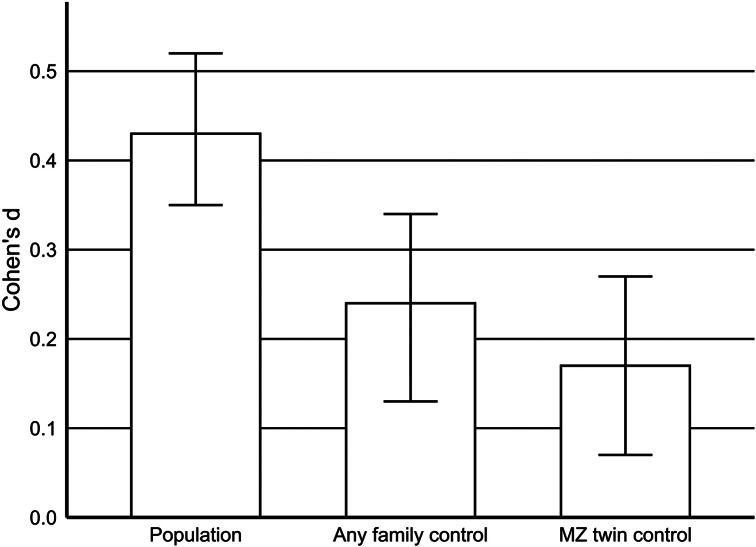
Mental health disparities (in Cohen’s d units, with 95% CIs) between heterosexuals and non-heterosexuals in naïve population estimates, with any family control (MZs, DZs, or siblings), and strict MZ twin controls. The data shown are based on separate random-effects meta-analyses of the three effect size types.

Figure 2 shows the mental health disparities for the three kinds of comparisons. Compared with the unadjusted comparison, the disparity was 47.5% lower (B_any family_ = −0.194, *SE* = 0.013, *p* = 0.001) if any family control was used, and 65.7% lower (B_MZ twin_ = −0.227, *SE* = 0.03, *p* = 0.001) between MZ twin pairs discordant for sexual orientation. Although the MZ twin estimate was lower than the simple family estimate, the difference was not statistically significant (*B* = −0.034, *p* = 0.272), likely owing to the small number of MZ twin estimates. Additional forest and funnel plots are available in the Supplementary materials Figures S1 and S2.

The very large sibling comparison analysis in (Xu, Rahman, Hiyoshi, & Montgomery, 2023a) was excluded from the main meta-analysis, for the reasons we gave earlier. If the study is included in the meta-analysis, the population estimate is slightly reduced (*B* = 0.386, *SE* = 0.043, *p* < 0.001), and the effect of any family control is smaller (*B* = −0.107, *SE* = 0.008, *p* < 0.001, 27.8% lower). However, while including the study does not significantly affect MZ control estimates, due to the lower population estimate, the reduction in MZ estimates is smaller (*B* = −0.207, *SE* = 0.029, *p* < 0.001, 53.7% lower).

## Discussion

Our results reveal that up to two-thirds of the correlation between non-heterosexual orientation and mental health disappears once family members discordant for sexual orientation but sharing familial causes (genes and shared environmental factors) are compared. This reduction identifies a substantial portion of this disparity which cannot be attributed to sexual minority stress. In light of this result, we conclude that much of the mental health disparities between heterosexual and non-heterosexual persons have been incorrectly attributed to sexual minority stress. Rather, a large proportion of the disparities is attributable to factors shared by both non-heterosexual and heterosexual persons who are genetically related and reared together. These factors cannot reflect sexual minority stress. Rather, they must reflect genetic or shared environmental factors present in the families of non-heterosexual persons and also affecting heterosexual members.

Importantly, non-heterosexual members of MZ twin pairs still exhibit elevated mental illness symptomatology compared with their heterosexual twins. This represents the intermediate case, whereby SMST is partially falsified, but not completely so. The remaining mental health disparity – about a third of what is seen in the population – must be caused by factors not shared by MZ twins reared together. Sexual minority stress is one candidate. Two other potential explanations of the remaining disparity consistent with findings of the current study have been discussed in a recent review of mental health of sexual minority individuals (Pachankis & Clark, 2024). Distinct Lifeways Theory emphasizes ways that the lives of non-heterosexual persons and heterosexual persons tend to differ. Examples include likelihood of family formation and distance from relatives. Narrative Possibilities Theory highlights potential iatrogenic harm of overemphasizing or exaggerating adversity in non-heterosexual persons’ lives. Paradoxically, identification with narratives of discrimination and victimhood may be especially likely in more tolerant societies where negative experiences of non-heterosexual persons are openly discussed. In contrast to SMST, the newer theories are consistent with otherwise anomalous empirical findings we have mentioned, including the lack of elevated rates of depression and anxiety in the U.S. Black population and the failure to find diminished rates of these problems in more tolerant cultures or eras.

### Limitations and future directions

There are limitations to the current meta-analysis. First, the demographic composition, definition of sexual orientation, and mental health measures in the included studies were heterogeneous, limiting our ability to draw conclusions about the effects of familial factors on specific mental health measures in specific demographic groups. Second, the analyses do not specifically identify genetic or shared environmental causes, although our results suggest evidence for genetic causes is higher. Third, although our analysis had adequate statistical power to confirm our primary hypothesis, parameter estimates were not estimated with great precision. Thus, we strongly encourage future studies employing gold-standard measures of mental health adjustment and including both siblings and MZ twins.

## Supporting information

10.1017/S0033291726105546.sm001 Ujma et al. supplementary material Ujma et al. supplementary material

## Supplementary material

The supplementary material for this article can be found at http://doi.org/10.1017/S0033291726105546.

## Long descriptions

### Figure 1. Long description

The flowchart is organized into three vertical phases: Identification, Screening, and Included.

1. Identification Phase:

- Top left: A grey box labeled Previous studies.

- Top right: An orange box labeled Identification of new studies via databases.

- Central flow: Records identified from databases (n = 453). An arrow points right to Records removed before screening: Duplicate records (n = 76).

- Far left: Other sources (n = 1) is shown in a grey box with a line bypassing the screening steps to the final inclusion box.

2. Screening Phase:

- Central flow: Records screened (n = 377). An arrow points right to Records excluded (n = 356).

- Central flow: Reports sought for retrieval (n = 21). An arrow points right to Reports not retrieved (n = 0).

- Central flow: Reports assessed for eligibility (n = 21). An arrow points right to Reports excluded: Duplicate database (n = 3) and Statistics unavailable (n = 2).

3. Included Phase:

- Central flow: New studies included in review (n = 16).

- Final step: Total studies included in review (n = 17), which combines the 16 new studies with the 1 study from other sources.

Navigate back to Figure 1..
