# Supplementary material for: How much is sexual minority stress confounded with familial causes? A systematic multilevel meta-analysis
Source: Psychol Med. 2026 Aug 10;56:e256. doi: 10.1017/S0033291726105546 (PMC13468942; doi:10.1017/S0033291726105546)
Supplement: Supplementary file 1 [file S0033291726105546sup001.docx]

**Supplementary figure S1**. Forest plots of the meta-analysis of sexual minority mental health disadvantage in population estimates, using any family controls, and strict MZ twin controls.

**Supplementary figure S2**. Forest plots of the meta-analysis of sexual minority mental health disadvantage in population estimates, using any family controls, and strict MZ twin controls.

**Supplementary text: Calculating effect sizes**

The meta-analysis uses Cohen’s d as the effect size of interest. Cohen’s d was calculated with three different sample specifications:

1) In the population (all heterosexuals vs. all non-heterosexuals, without regard for family membership)
2) Any family control (heterosexual vs. non-heterosexual members of the same family, pooling estimates of various relationship degrees such as siblings, DZ and MZ twins)
3) MZ twin control (between MZ twins discordant for sexual orientation).

Whenever possible, Cohen’s d was calculated directly from means and standard deviations.

Odds ratios were converted to Cohen’s d using the following formula:

d = (ln(OR) × √3) / π

One study [(Xu et al. 2023)](https://sciwheel.com/work/citation?ids=17936455&pre=&suf=&sa=0&dbf=0) reported a sibling comparison using hazard ratios instead of odds ratios. Hazard and odds ratios can be different if the rate of hazard is not constant, or if the events are not rare. The rate of hazard cannot be estimated from this study, but Table 1 reports the rate of events, which are relatively high and the estimated odds ratios are substantially different from the raw hazard ratios reported on Figure 2, Model 1. Therefore, this study was omitted from the main meta-analysis. However, due to its substantial size, it was re-included in an exploratory step, with hazard ratios converted using the formula used for odds ratios.

Pearson correlations were converted using the following formula:

d = 2r / √(1 - r²)

Several studies [(Olakunle Ayokunmi Oginni et al. 2023; Olakunle A Oginni et al. 2023; Xu et al. 2023; Brendan P Zietsch et al. 2011; B P Zietsch et al. 2012)](https://sciwheel.com/work/citation?ids=14250591,17312638,1855246,1855397,17936455&pre=&pre=&pre=&pre=&pre=&suf=&suf=&suf=&suf=&suf=&sa=0,0,0,0,0&dbf=0&dbf=0&dbf=0&dbf=0&dbf=0) reported biometric estimates (genetic and nonshared environmental correlations between sexual orientation and mental health) instead of a direct within-twin pair comparison. With this method, the population correlation r can be described as:.

r = √(h1²) × √(h2²) × rA + √(e1²) × √(e2²) × rE

where h² and e² are heritabilities and nonshared environmentalities of trait 1 and 2, and rA, rE are the genetic and environmental cross-trait correlations. In other words, the population correlation between the two traits exists as a sum of a genetic and an environmental path. Within MZ pairs, only the environmental path operates, the genetic path is zero. Within DZ pairs, in addition to this 50% of the genetic path also operates. Therefore, MZ correlations can be estimated from just the second term, and DZ correlations as the second term plus half the value of the first term. Within-family correlations, in turn, can be estimated as the population-size weighted average of MZ and DZ correlations. This process can also be used if the study is based on other kinships, such as biological and adoptive siblings [(Xu et al. 2023)](https://sciwheel.com/work/citation?ids=17936455&pre=&suf=&sa=0&dbf=0). Within-MZ and within-family Pearson correlations were estimated using this method, and converted to Cohen’s d using the formula above.

**References**

[Oginni, Olakunle A, Lim, K. X., Rahman, Q., Jern, P., Eley, T. C., & Rijsdijk, F. V. (2023). Bidirectional Causal Associations Between Same-Sex Attraction and Psychological Distress: Testing Moderation and Mediation Effects. *Behavior Genetics*, *53*(2), 118–131. https://doi.org/10.1007/s10519-022-10130-x](https://sciwheel.com/work/bibliography/14250591)

[Oginni, Olakunle Ayokunmi, Lim, K. X., Purves, K. L., Lu, Y., Johansson, A., Jern, P., & Rijsdijk, F. V. (2023). Causal Influences of Same-Sex Attraction on Psychological Distress and Risky Sexual Behaviors: Evidence for Bidirectional Effects. *Archives of Sexual Behavior*, *52*(3), 1213–1228. https://doi.org/10.1007/s10508-022-02455-9](https://sciwheel.com/work/bibliography/17312638)

[Xu, Y., Rahman, Q., Hiyoshi, A., & Montgomery, S. (2023). Same-Sex Marriage and Common Mental Health Diagnoses: A Sibling Comparison and Adoption Approach. *Journal of Sex Research*, *60*(5), 585–595. https://doi.org/10.1080/00224499.2022.2120597](https://sciwheel.com/work/bibliography/17936455)

[Zietsch, Brendan P, Verweij, K. J. H., Bailey, J. M., Wright, M. J., & Martin, N. G. (2011). Sexual orientation and psychiatric vulnerability: a twin study of neuroticism and psychoticism. *Archives of Sexual Behavior*, *40*(1), 133–142. https://doi.org/10.1007/s10508-009-9508-4](https://sciwheel.com/work/bibliography/1855246)

[Zietsch, B P, Verweij, K. J. H., Heath, A. C., Madden, P. A. F., Martin, N. G., Nelson, E. C., & Lynskey, M. T. (2012). Do shared etiological factors contribute to the relationship between sexual orientation and depression? *Psychological Medicine*, *42*(3), 521–532. https://doi.org/10.1017/S0033291711001577](https://sciwheel.com/work/bibliography/1855397)
